# Selected traditional Chinese herbal medicines for the treatment of atopic dermatitis - research progress on the effect and mechanism of actions

**DOI:** 10.3389/fphar.2025.1553251

**Published:** 2025-03-26

**Authors:** Lingjie Zhang, Hangjuan Lin, Ninggang Chen, Suyan Zhu, Ying Hu

**Affiliations:** ^1^ College of Biological and Environmental Sciences, Zhejiang Wanli University, Ningbo, Zhejiang, China; ^2^ Ningbo Municipal Hospital of Traditional Chinese Medicine (TCM), Affiliated Hospital of Zhejiang Chinese Medical University, Ningbo, Zhejiang, China; ^3^ College of Pharmacy, Zhejiang Pharmaceutical University, Ningbo, Zhejiang, China; ^4^ College of Pharmacy, The First Affiliated Hospital of Ningbo University, Zhejiang, China

**Keywords:** atopic dermatitis, traditional Chinese medicine, therapeutic drugs, action mechanism, review

## Abstract

Atopic dermatitis (AD) is a common chronic, recurrent, inflammatory skin disease characterized by pruritus, lichen-like changes and dry skin. Due to the complex pathogenesis of AD, its mechanism is primarily associated with genetic, skin barrier dysfunction, environmental, and immune factors. AD has been routinely treated with glucocorticoids, antihistamines, local immunomodulators, biological agents, and small molecules; however, the side effects are significant, and the treatment efficacy is limited. In recent years, traditional Chinese medicine (TCM) has gradually been widely used in the treatment of AD. Many studies have shown that TCM mainly regulates inflammatory cytokines, gut microbiota and the immune system. Therefore, it plays a crucial role in the treatment of AD. The treatment of atopic dermatitis using TCM is characterized by targeting multiple pathways and multiple targets, and it demonstrates significant therapeutic effects. This paper reviews the pathogenesis of AD and reports the efficacy of TCM on AD (including TCM prescription, single TCM, treatment of TCM metabolites), which provides a theoretical basis for TCM treatment of AD. TCM has certain therapeutic effects on AD. It can alleviate and treat AD in various ways. We should base our differentiation on syndrome differentiation and treatment differentiation. With the help of modern medicine, the clinical efficacy of TCM in treating AD can be improved.

## 1 Introduction

**FIGURE 1:**
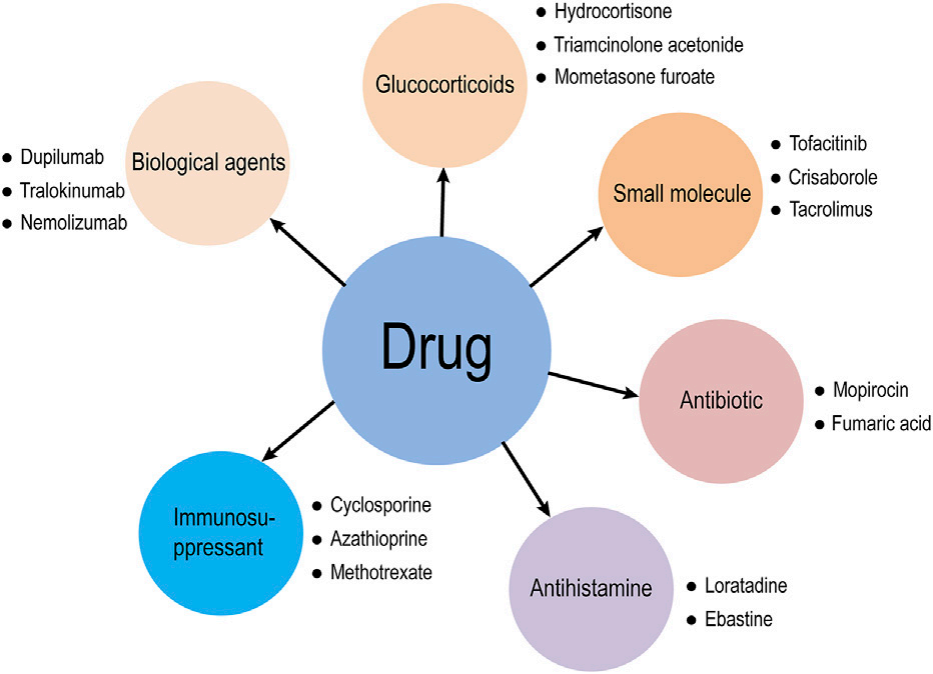
Drugs for clinical treatment of AD.

Atopic dermatitis (AD) is a chronic inflammatory skin disease with a genetic predisposition related to the environment, skin barrier, immune system, and bacterial infection. AD is a global health problem, and although its pathogenesis involves multiple factors such as genetic predisposition and environmental triggers, it is not yet fully understood (Zhang et al., 2022a). The prevalence of AD is increasing worldwide, affecting approximately 20% of children and 3% of adults (Zeng et al., 2019). It imposes a significant psychosocial burden on patients because it creates an unsightly appearance and functional limitations. It also increases the likelihood of developing diseases, including asthma, arthritis, allergic rhinitis, food allergies and other inflammatory diseases, including mental disorders (Hemrajani et al., 2022). The purpose of treatment is to alleviate symptoms, prevent recurrent attacks, and reduce risks. Clinical medications for AD include glucocorticoids, calcineurin inhibitors, antibiotics, antihistamines, targeted biological agents, small molecules, and immunosuppressants (Figure 1). Many studies have shown a lack of appropriate, safe and effective AD treatments (Liu W. et al., 2022). Over recent years, a growing number of people are paying attention to the more effective and safe use of traditional Chinese medicine (TCM) to treat AD.

## 2 Pathogenesis of AD

The etiology and pathogenesis of the disease are not yet fully understood (Jia, 2020), and it may have an inseparable relationship with genetics, skin barrier dysfunction (Chong et al., 2022), environmental factors (Gu et al., 2023), and also involves immunology, neuropsychiatric factors, and gut microbiota (Liu Y. et al., 2022).

### 2.1 Genetic factors and skin barrier damage

AD often occurs in individuals with a family history of allergies, including asthma and allergic rhinitis (Williamson et al., 2020). Genetic factors play a significant role in AD pathogenesis, as evidenced by twin studies showing high heritability (72%–86% in monozygotic twins vs. 21%–23% in dizygotic twins) (Thomsen et al., 2007). Recent studies have identified multiple genes potentially associated with AD susceptibility across diverse populations (Chen and Chen, 2022), with at least 34 genetic loci and 46 genes linked to AD risk globally (Schmidt and de Guzman Strong, 2021). The most well-characterized genetic association involves the filaggrin gene (FLG). The protein encoded by this gene is crucial for maintaining the integrity of the epidermal barrier (Naeem et al., 2017). FLG mutations impair skin barrier function, and emerging evidence highlights the significance of epigenetic mechanisms regulating FLG expression in disease pathogenesis (Yang et al., 2020), FLG levels are generally reduced in AD patients, which destroys the structure of the stratum corneum, increases the permeability of the cell membrane, and causes a large loss of epidermal water, which may be related to the mutation of FLG gene (Drislane and Irvine, 2020), therefore, FLG gene mutations, which lead to dysfunction, are among the most significant pathogenic factors in AD (Smieszek et al., 2020). The skin’s primary function resides in establishing an efficient barrier between an organism’s internal milieu and external environment. Consequently, skin serves as a critical interface providing both protective and structural support for the organism it encapsulates (Miura et al., 2025). Barrier dysfunction initiates a pathogenic cascade wherein environmental stimuli activate keratinocytes to release alarmins (such as thymic stromal lymphopoietin), triggering type 2 immune responses through interleukin production. This mechanism exacerbates barrier defects and potentiates allergic inflammation (van den Bogaard et al., 2023).

### 2.2 Environmental factors

The influence of environment is related to the pathogenesis of AD, and many studies have shown that environmental factors can exacerbate the symptoms of AD patients (Kantor and Silverberg, 2017). The onset of AD may change the skin penetration through the stimulation of various substances in the environment to the skin, resulting in the impairment of skin barrier function, making it easier for external stimuli to enter the skin and trigger inflammatory reactions (Thibault Greugny et al., 2023). This may be related to the abnormal synthesis and secretion of skin barrier proteins such as keratin and intercellular lipids. Exposure to dynamic environmental changes may induce or cause disease in susceptible populations. There are complex interactions between different environmental factors, including personal use of personal care products and exposure to climate, pollution, food, and other extrinsic factors (Narla and Silverberg, 2020; Lai et al., 2023; Mailepessov et al., 2024).

### 2.3 Diseases of the immune system

**FIGURE 2:**
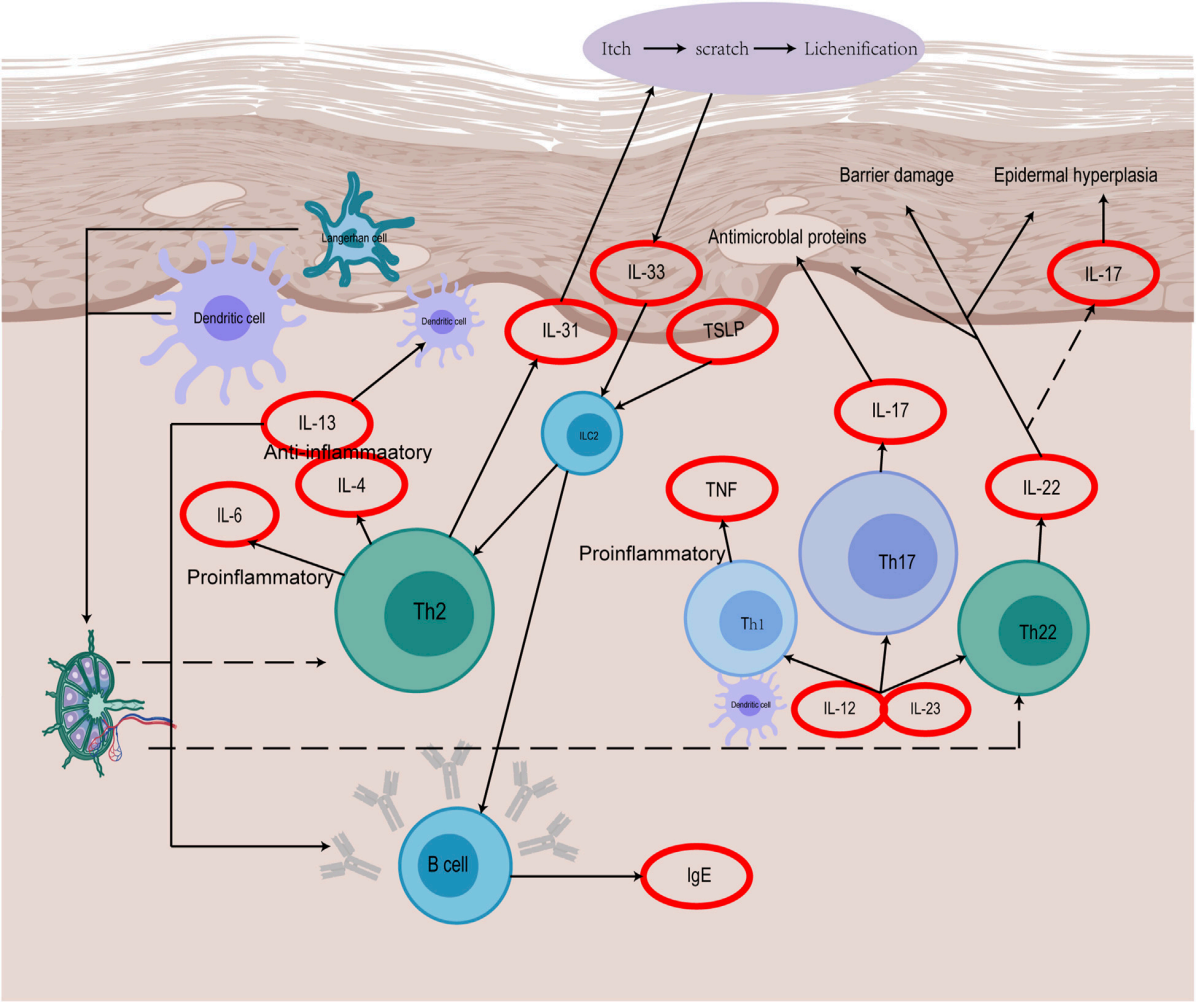
The immune mechanism of AD.

Immune dysfunction is the central link in the pathogenesis of AD (Figure 2). Keratinocytes (KCs) are the regulatory cells of the innate immune response. When the expression of KCs is abnormal, AD-related symptoms are triggered on the surface of the skin, and there are also many abnormalities in immune factors (De Bruyn Carlier et al., 2021), such as KCs, dendritic cells (DCs), mast cells (MCs) and Th cells. These are the sources of the pathogenesis of AD. Previous studies on AD have been focused predominantly on Th2-mediated immune responses. The imbalance of Th1/Th2 immunity caused by cytokines such as IL-4 causes skin inflammation and damages skin barrier function (Oh et al., 2021; Sroka-Tomaszewska and Trzeciak, 2021). According to recent studies, ad is obviously Th2/Th22 centered with the overexpression of Th1 and Th17 cell related cytokines (Li H. et al., 2021), and the additional activation pathways of Th22 and Th17 cytokines through the release of IL-17, IL-19, and IL-22, as well as the role of regulating lymphocytes as another mechanism of AD have been widely discussed (Brunner et al., 2017; Gatmaitan and Lee, 2023).

### 2.4 Neural and psychiatric factors

The core symptom of AD is pruritus, which is primarily mediated by neurogenic signaling pathways. Th2 cytokines can directly activate sensory neurons and mediate itch signals (Kwatra et al., 2022). Transient receptor potential channels play a key role in inflammation and nerve sensitization, leading to a decrease in itch threshold (Aldossary et al., 2024). Sensory nerve endings in the skin release neuropeptides, trigger “neurogenic inflammation,” promote mast cell degranulation, and release inflammatory mediators such as histamine and TNF-α (Xu et al., 2020), activate keratinocytes, secrete cytokines such as IL-33 and TSLP, and amplify Th2 immune response (Ikai et al., 2023). At the same time, inflammatory factors further affect the central nervous system through the “brain skin axis” (Chitnis and Weiner, 2017). The density of nerve fibers increases in the skin of AD patients, especially itch related nerves, resulting in enhanced itch perception. Overexpression of nerve growth factor promotes nerve fiber proliferation and sensitization (Tominaga et al., 2009). Nerve and mental factors play a key role in the occurrence and chronicity of AD through complex neuroimmune interactions, itch signaling, and psychophysiological feedback loops. Future research needs to further analyze the molecular mechanism of “brain skin axis” to provide basis for individualized treatment.

### 2.5 Intestinal floras

Studies have shown that early life is a critical period for appropriate immune responses to microorganisms, which may be a critical period for initiating microbiome interventions (Koh et al., 2022). In recent years, with the concept of the “gut skin” axis being proposed (Zhang et al., 2024), the imbalance of gut microbiota abundance is considered to be another important factor in the occurrence of AD. The status of gut microbiota greatly affects the differentiation of naive T cells into other types of Th cells, regulating the immune system of the body and skin (Markowiak-Kopeć and Śliżewska, 2020). Further studies on the interaction between human microbiota and AD will provide new targets for the treatment of AD in the future.

## 3 TCM for AD treatment

The concept of AD does not have a complete counterpart in TCM, ancient physicians, who formerly referred to AD as eczema, wet sores, wet tinea, blood wind sores, or four-curved wind sores, believed that the onset of AD was related to the environment, diet, and daily life. It was caused by internal factors such as Heart Fire and Spleen Dampness, and external factors such as Wind Evil.

### 3.1 Five elements and five Yin/Yang in TCM

TCM interprets disease pathogenesis through a holistic lens that integrates the Five Elements (Wu Xing), Yin-Yang organ relationships, and exogenous/endogenous pathogenic factors. These theories collectively explain systemic imbalances and guide therapeutic strategies, such as those for AD. While modern dermatology focuses on molecular pathways, TCM emphasizes the interplay between organ networks and environmental influences. Table 1 is a structured summary of these foundational concepts and their clinical relevance.

**TABLE 1:** Five elements, Yin-Yang organ Pairs, and pathogenic factors.

Theory	Key components	Clinical correlation
Five Elements	Wood: Liver/Gallbladde; Governs Qi flow, emotional regulation Fire: Heart/Small Intestine; Regulates blood circulation Earth: Spleen/Stomach; Controls digestion and dampness Metal: Lung/Large Intestine; Manages skin and immunity Water: Kidney/Bladder; Governs growth and hydration	Chronic stress (Wood/Liver dysfunction) → Qi stagnation → eczema flares Heart Fire exacerbates erythema and itching Spleen deficiency → dampness accumulation → oozing lesions Lung Qi deficiency → dry, flaky skin (impaired barrier function) Kidney Yin deficiency leads to chronic dryness and lichenification
Yin-Yang Organ Pairs	Yin (Zang): Solid, nutrient-storing organs (Liver, Spleen) Yang (Fu): Hollow, transport-focused organs (Stomach, Gallbladder)	Yin deficiency (Spleen) → poor skin hydration Yang excess (Stomach Heat) → inflammatory erythema
Pathogenic Factors	External: Wind (itching), Damp (exudation), Heat (redness), Dryness (scaling) Internal: Emotional stress (Liver Qi stagnation), Blood deficiency	Acute AD: Wind-Heat-Damp invasion → pruritic, erythematous plaques Chronic AD: Blood deficiency → xerosis; Qi stagnation → lichenification

### 3.2 Research progress in the treatment of AD with TCM

**FIGURE 3:**
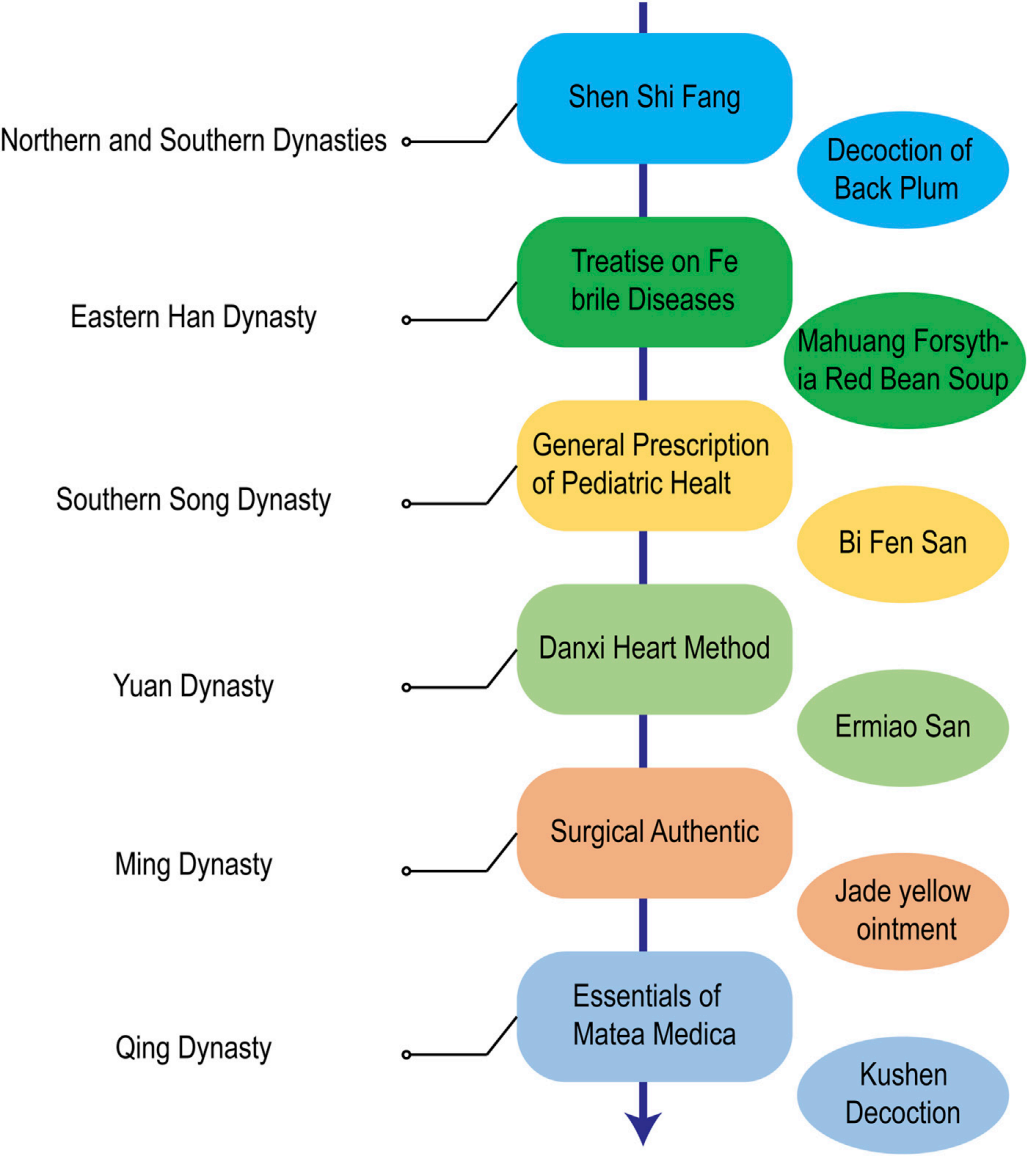
Historical evolution of AD treatment with TCM in China.

**FIGURE 4:**
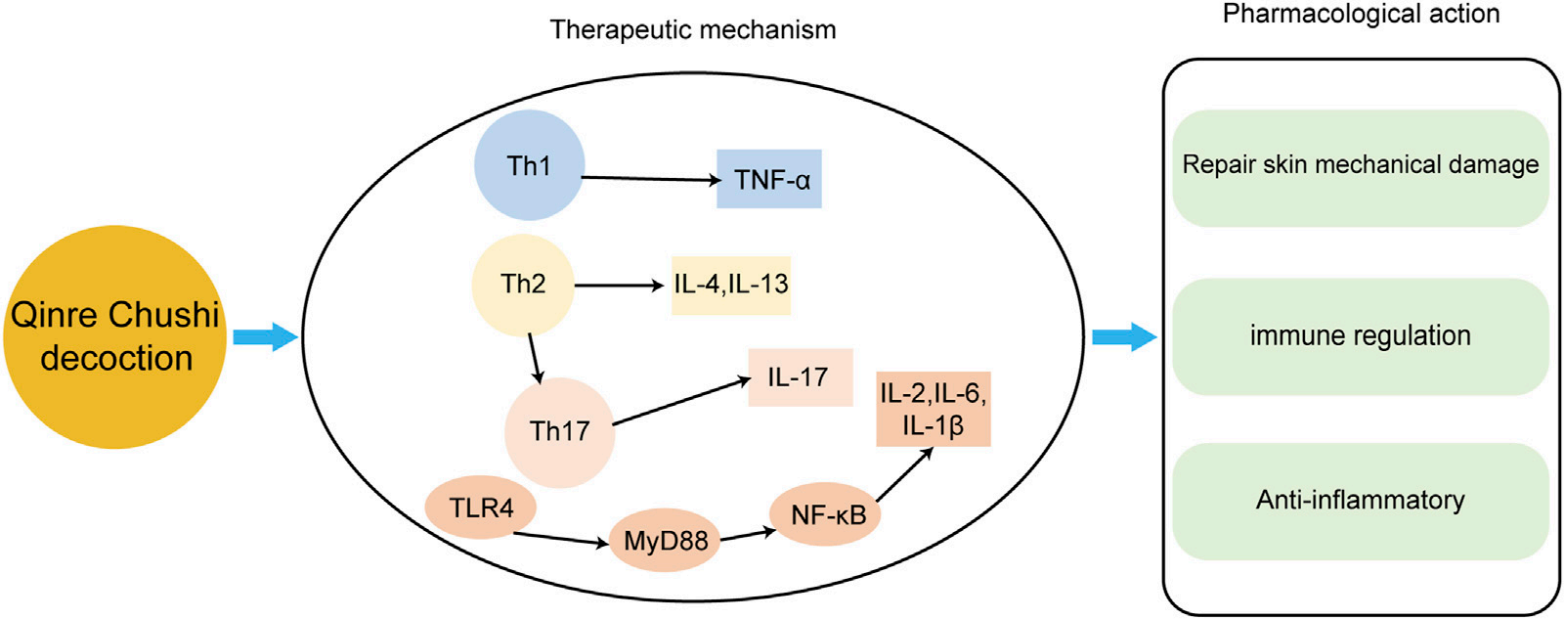
The main mechanisms of Qingre Chushi decoction against AD.

Although AD is not accurately and in detail recorded in TCM, the discussions in ancient Chinese medicine books have important reference value and guiding significance for today’s understanding and treatment of AD (Figure 3). It is recorded in Shen Shi Fang of the Northern and Southern Dynasties that the decoction of black plum can Dry Damp ringworm Zhang proposed in Treatise on Febrile Diseases in the Eastern Han Dynasty that Ephedra, Forsythia and Chixiaodou Decoction could clear away Heat Toxin, Dispel Dampness and Streng Spleen. During the Southern Song Dynasty, there was a record in the General Prescription of Pediatric Healt that Diyu treated facial ulcers, redness, swelling, and pain. During the Yuan Dynasty, Zhu proposed the use of Ermiao Powder in the Danxi Heart Method to treat inflammation, redness, swelling, and exudation caused by dampness and heat. During the Ming Dynasty, Chen proposed the use of Xiaofeng Powder for the treatment of eczema in his Surgical Authentic. During the Qing Dynasty, Wang recorded in his Essentials of Matea Medica that Kushen was used to Dry Dampness and remove heat. Nowadays, TCM prescription is the most common TCM treatment method, which allows the easy adjustment of dosage and drug composition according to the conditions (Figure 4). In recent years, some external creams and moisturizing agents based on active substances found in TCM have been commonly used in the treatment of AD and have achieved satisfactory results. TCM ointment is a proprietary medicine with a long history of treating diseases. It is a semiliquid thick paste that is created by preparing a large decoction through the addition of certain excipients after concentration. Umbilical compression treatment and acupuncture treatment have produced unexpected effects on many chronic medical diseases, both of which belong to the classical treatment methods of TCM. TCM baths are a classic TCM method for treating skin diseases and are also an effective method of treating intractable skin diseases. The use of medicinal baths makes the treatment method more scientific, safe, environmentally friendly, and humanized.

### 3.3 Therapeutic effect of TCM on AD

#### 3.3.1 TCM prescription

TCM preparations have unique advantages in the treatment of AD. Experimental studies have found that TCM preparations have multiple targets for the treatment of AD, and most of them have been applied in clinical practice with demonstrated favorable outcomes. Among them is the classic botanical drug formula Angelica Yinzi (AYZ) comes from the Yan’s Jisheng Formula during the Southern Song Dynasty (Yan’s Jisheng Formula volume VI). The efficacy of AYZ has been scientifically and clinically proven and there have been no serious adverse reactions. The AYZ formula contains 11 different botanical drug: Dang Gui (*Angelica sinensis* (Oliv.) Diels [Apiaceae]), Shao Yao (*Paeonia lactiflora* Pall. [Paeoniaceae]), Chuan Xiong (*Conioselinum anthriscoides ‘Chuanxiong’* [Apiaceae]), Di Huang (*Rehmannia glutinosa* (Gaertn.) Libosch. ex DC. [Orobanchaceae]), Ji Li (*Tribulus cistoides* L. [Zygophyllaceae]), Fang Feng (*Saposhnikovia divaricata* (Turcz. ex Ledeb.) Schischk.), Jing Jie (*Nepeta tenuifolia* Benth. [Lamiaceae]), He Shou Wu (*Reynoutria multiflora* (Thunb.) Moldenke [Polygonaceae]), Huang QI (*Astragalus mongholicus* Bunge [Fabaceae]), Gan Cao (*Glycyrrhiza glabra* L. [Fabaceae]), ShengJiang (*Zingiber officinale* Roscoe [Zingiberaceae]). Wang et al. (2021) used network pharmacology methods to suggest AYZ may be able to intervene in AD by regulating the MAPK/NF-κB signalling pathways. Liu et al. (see Table 2) treated DNCB-induced AD-like mice with AYZ and found that AYZ decreased the serum IgE level, decreased the epidermal thickness of AD-like lesion skin, and AYZ can downregulate inflammatory factors TNF-α and IL-1β by inhibiting the MAPK/NF-κB signalling pathways. The treatment downregulates the expression of NLRP3, which significantly decreases the activation of inflammatory bodies in NLRP3, improves the symptoms of AD (Liu W. et al., 2022).

**TABLE 2:** The Source species of TCM involved in this paper.

No.	Chinese name	Family name	Latin name	Source species
1	Dang Gui	Apiaceae	*Angelicae Sinensis Radix*	*Angelica sinensis* (Oliv.) Diels [Apiaceae]
2	Shao Yao	Paeoniaceae	Paeonia lactiflora Pall	*Paeonia lactiflora* Pall. [Paeoniaceae]
3	Chuan Xiong	Apiaceae	*Ligusticum chuanxiong* Hort.	*Conioselinum anthriscoides* ‘Chuanxiong’ [Apiaceae]
4	Di Huang	Ledangae	*Rehmanniae Radix Praeparata*	*Rehmannia glutinosa* (Gaertn.) Libosch. ex DC. [Orobanchaceae]
5	Ji Li	Tribulus	*Tribulus terrestris* Linnaeus	*Tribulus terrestris* L. [Zygophyllaceae]
6	Fang Feng	Apiaceae	*Saposhnikoviae Rqadix*	*Saposhnikovia divaricata* (Turcz. ex Ledeb.) Schischk. [Apiaceae]
7	Jing Jie	Labiatae	*Schizonepetae Herba*	*Nepeta tenuifolia* Benth. [Lamiaceae]
8	He Shou Wu	Polygonaceae	*Polygoni Multiflori Radix*	*Reynoutria multiflora* (Thunb.) Moldenke [Polygonaceae]
9	Huang Qi	Leguminosae	*Astragali Radix*	*Astragalus mongholicus* Bunge [Fabaceae]
10	Gan cao	Leguminosae	*Glycyrrhizae Radix et Rhizoma*	*Glycyrrhiza glabra* L. [Fabaceae]
11	ShengJiang	Curcumaceae	*Zingiber officinale* Roscoe	*Zingiber officinale* Roscoe [Zingiberaceae]
12	Ma Huang	Ephedraceae	*Ephedra Herba*	*Ephedra sinica* Stapf [Ephedraceae]
13	Lian Qiao	Oleaceae	*Forsythia suspensa*	*Forsythia suspensa* (Thunb.) Vahl [Oleaceae]
14	Xing Ren	Rosaceae	*Prunus armeniaca*	*Prunus amygdalus* Batsch [Rosaceae]
15	Chi Dou	Leguminosae	*Vigna umbellata*	*Vigna umbellata* (Thunb.) Ohwi & H.Ohashi [Fabaceae]
16	Da Zao	Rhamnaceae	*Jujubae Fructus*	*Ziziphus jujuba* Mill. [Rhamnaceae]
17	Sang Bai Pi	Moriaceae	*Morus alba* L.	*Morus indica* L. [Moraceae]
18	Zi Hua Di Ding	Violaceae	*Viola yedoensis Makino*	*Viola hamiltoniana* D.Don [Violaceae]
19	Ku Shen	Leguminosae	*Sophorae Flavescentis Radix*	*Sophora flavescens* Aiton [Fabaceae]
20	Bai Xian	Rutaceae	*Dictamnus albus*	*Dictamnus dasycarpus* Turcz. [Rutaceae]
21	Jin Yin Hua	Lonicera	*Lonicera japonica Thunb*	*Lonicera japonica* Thunb. [Caprifoliaceae]
22	Huang Bai	Rutaceae	*Cortex Phellodendri*	*Phellodendron amurense* Rupr. [Rutaceae]
23	Pu Gong Yin	Asteraceae	*Taraxacum mongolicum*	*Taraxacum mongolicum* Hand.-Mazz. [Asteraceae]
24	Da Hang	Polygonaceae	*Radix et Rhizoma Rhei*	*Rheum palmatum* L. [Polygonaceae]
25	Ku Di Ding	Papaveridae	*Corydalis Bungeanae Herba*	*Corydalis bungeana* Turcz. [Papaveraceae]
26	Huang Qin	Labiatae	*Scutellariae Radix*	*Scutellaria baicalensis* Georgi [Lamiaceae]
27	Di Yu	Rosaceae	*Radix Sanguisorbae*	*Sanguisorba officinalis L. [Rosaceae]*
28	Ma Chi Xian	Portulaceae	*Portulacae Herba*	*Portulaca oleracea* L. [Portulacaceae]
29	Cang Shu	Asteraceae	*Atractylodes*	*Atractylodes lancea* (Thunb.) DC. [Asteraceae]
30	Yin Yang Huo	Berberidaceae	*Epimedium chlorandrum Stearn*	*Epimedium brevicornu* Maxim. [Berberidaceae]
31	San Qi	Araliaceae	*Notoginseng Radix*	*Panax notoginseng* (Burkill) F.H.Chen [Araliaceae]
32	Tu Fu Ling	Smilacaceae	*Smilacis Glabrae Rhixoma*	*Smilax glabra* Roxb. [Smilacaceae]
33	Che Qian Cao	Plantaginaceae	*Plantaginis Herba*	*Plantago asiatica* L. [Plantaginaceae]
34	Ban Lan Gen	Brassicaceae	*Isatidis Radix*	*Isatis tinctoria* L. [Brassicaceae]
35	Huang Shan Yao	Dioscoreaceae	*Dioscorea panthaiccae rhizoma*	*Dioscorea panthaica* Prain & Burkill [Dioscoreaceae]
36	Ze Xie	Alismaceae	*Alisma Orientale*	*Alisma plantago-aquatica subsp. orientale* (Sam.) Sam. [Alismataceae]
37	Mu Dan Pi	Ranunculaceae	*Paeoniae Radix Moutan*	*Paeonia × suffruticosa* Andrews [Paeoniaceae]
38	Bo He	Lamiaceae	*Menthae Herba*	*Mentha canadensis* L. [Lamiaceae]
39	Bai Shu	Asteraceae	*Atractylodes Macrocephala* Koidz.	*Atractylodes macrocephala* Koidz. [Asteraceae]
40	BaiShao	Paeoniaceae	*Paeoniae Radix* Alba	*Paeonia lactiflora* Pall. [Paeoniaceae]
41	Bai zhi	Apiaceae	*Angelica dahurica*	*Angelica dahurica* (Hoffm.) Benth. & Hook.f. ex Franch. & Sav. [Apiaceae]
42	Zi Ding Xiang	Oleaceae	*Syringa oblata*	*Syringa oblata* Lindl. [Oleaceae]
43	Ji Xue Teng	Fabaceae	*Spatholobus Suberectus* Dunn	*Spatholobus suberectus* Dunn [Fabaceae]
44	She Chuang Zi	Apiaceae	*Cnidii Fructus*	*Cnidium monnieri* (L.) Cusson [Apiaceae]
45	Lu Hui	Asphodelaceae	*Aloe*	*Aloe vera* (L.) Burm.f. [Asphodelaceae]
46	Yi Ren	Poaceae	*Coicis Semen*	*Coix lacryma-jobi* L. [Poaceae]
47	Hai Tong Pi	Leguminosae	*Cortex Erythrinae*	*Erythrina variegata* L. [Fabaceae]
48	Zi Cao	Boraginaceae	*Lithospermum Erythrorhizon*	*Lithospermum erythrorhizon* Siebold & Zucc. [Boraginaceae]
49	Ya Zui Hua	Acanthaceae	*Justicia adhatoda* L.	*Justicia adhatoda* L. [Acanthaceae]
50	Hu Zhang	Polygonaceae	*Polygoni Cuspidati Rhizoma Et Radix*	*Reynoutria japonica* Houtt. [Polygonaceae]
51	Deng Zhan Hua	Asteraceae	*Erigeron breviscapus*	*Erigeron breviscapus* (Vaniot) Hand.-Mazz. [Asteraceae]
52	Mu Ju	Asteraceae	*Matricaria recutita*	*Matricaria chamomilla* L. [Asteraceae]
53	Huang Lian	Ranunculaceae	*Coptidis Rhizoma*	*Coptis trifolia* (L.) Salisb. [Ranunculaceae]

In addition, the Mahuang Lianqiao Chixiaodou Decoction (MLCD) incorporated in “Treatise on Cold Injury and Miscellaneous Diseases” is a classic TCM formula that is believed to resolve external (i.e., skin surface) and internal (i.e., visceral dysfunction) symptoms and signs by Expelling Wind, Moisture, and Heat Pathogens to Strengthen Spleen, reduce fever, and Expel Moisture. Recently, Yuan et al. showed that modified MLCD exhibits promising results in the treatment of AD, and significantly improves the pathological state of AD patients, alleviates itching, and reduces the recurrence rate. The mechanism may be related to regulating Th1/Th2 immune imbalance (Yuan et al., 2022).

There are also some classic prescriptions, such as the Viola yedoensis Makino anti-itching metabolite, the Huangbai liniment and Qingxue jiedu formulation, which can be used to treat AD (Table 3). They come from ancient books and modern clinical treatment cases, and are worth learning and further development and utilization.

**TABLE 3:** The effects of TCM formulas on AD.

Name	Botanical drugs	Evaluation model	The effect mechanism	The literature
Angelica Yinzi Decoction	Dang Gui (*Angelica sinensis* (Oliv.) Diels), Shao Yao (*Paeonia lactiflora* Pall.), Chuan Xiong (*Conioselinum anthriscoides ‘Chuanxiong*’), Di Huang (*Rehmannia glutinosa* (Gaertn.) Libosch. ex DC.), Ji Li (*Tribulus terrestris* L.), Fang Feng (*Saposhnikovia divaricata* (Turcz. ex Ledeb.) Schischk.), Jing Jie (*Nepeta tenuifolia* Benth.), He Shou Wu (*Reynoutria multiflora* (Thunb.) Moldenke), Huang Qi (*Astragalus mongholicus* Bunge), Gan Cao (*Glycyrrhiza glabra* L.), Sheng Jiang (*Zingiber officinale* Roscoe)	DNCB - induced AD in mice	Inhibiting the activation of the NLRP3 inflammasome and the MAPKs/NF-κB signaling	Liu et al. (2022a)
Qingre Chushi decoction	Long Dan Cao (*Gentiana scabra* Bunge), Bai Mao Gen (*Imperata cylindrica* (L.) Raeusch.), Sheng Di Huang (*Rehmannia glutinosa* (Gaertn.) Libosch. ex DC.), Da Qing Ye (*Isatis tinctoria subsp. tinctoria*), Che Qian Cao (*Plantago asiatica* L.), Sheng Shi Gao (*Gypsum Fibrosum*),Huang Qin (*Scutellaria baicalensis* Georgi),Hua Shi (*Talcum*), Gan Cao (*Glycyrrhiza glabra* L.).	DNFB - induced AD in mice	Alleviate inflammatory reactions, reduce the number of mast cells, and regulate Th1/Th2 balance	Meng (2021)
Mahuang Lianqiao Chixiaodou decoction	Ma Huang (*Ephedra sinica* Stapf), Lian Qiao (*Forsythia suspensa* (Thunb.) Vahl), Xing Ren (*Prunus armeniaca* L.), Chi Xiao Dou (*Vigna umbellata* (Thunb.) Ohwi & H.Ohashi), Da Zao (*Ziziphus jujuba* Mill.), Sang Bai Pi (*Morus indica* L.), Sheng Jiang (*Zingiber officinale* Roscoe), Gan Cao (*Glycyrrhiza glabra* L.).	DNFB - induced AD in mice	Significantly reduced therelative expression of IL-4 and TSLP mRNA in the lesion tissues,as well as the serum level of IL-4	Yuan et al. (2022)
Shen Chan decoction	Dang Shen (*Codonopsis pilosula* (Franch.) Nannf.), Bai Zhu (*Atractylodes Macrocephala* Koidz.), Fu Ling (*Poria Cocos* (Schw.) Wolf.), Shan Yao (*Dioscorea oppositifolia* L.), Yi Yi Ren (*Coix lacryma-jobi var. ma-yuen* (Rom.Caill.) Stapf), Huang Qi (*Astragalus mongholicus* Bunge.), Da Zao (*Ziziphus jujuba* Mill.), Gan Cao (*Glycyrrhiza glabra* L.).	DNCB - induced AD in mice	Balance Th1/Th2 cytokines and inhibit H1R/PAR-2/TRPV1 Itch signal transduction; adjust microorganisms in the gut	Zhang et al. (2024)
Viola yedoensisMakino anti-itching metabolite	Zi Hua Di Ding (*Viola hamiltoniana* D.Don), Ku Shen (*Sophora flavescens* Aiton), Bai Xian (*Dictamnus dasycarpus* Turcz.)	DNCB - induced AD in mice	Inhibiting the inflammatory mediator productions and blocking mast cell degranulationviasuppressing Syk mediated NF-κB pathway reduces the levels of inflammatory factors by activating JAK2/STAT3 signaling pathway and promoting M2 macrophages polarization	Zeng et al. (2021)
Huangbai Liniment	Lian Qiao (*Forsythia suspensa* (Thunb.) Vahl), Jin Yin Hua (*Lonicera japonica* Thunb.), Huang Bai (*Phellodendron amurense* Rupr.), Pu Gong Yin (*Taraxacum sect. Taraxacum* F.H.Wigg.), Wu Gong (*centipede*)	DNCB - induced AD in mice	Reduced the expression ofproinflammatory cytokines, including IL-1β, IL-4, IFN-γ, IL-13,and IL-17, and increased the expression of anti-inflammatorycytokine IL-10	Zheng et al. (2021)
Qingxue jiedu formulation	Da Hang (*Rheum palmatum* L), Jing Jie (*Nepeta tenuifolia* Benth.), Pu Gong Ying (*Taraxacum sect. Taraxacum* F.H.Wigg.), Fang Feng (*Saposhnikovia divaricata* (Turcz. ex Ledeb.) Schischk.), Ku Di Ding (*Corydalis bungeana* Turcz.), Huang Qin (*Scutellaria baicalensis* Georgi), Lian Qiao (*Forsythia suspensa* (Thunb.) Vahl), Gan Cao (*Glycyrrhiza glabra* L.), Di Huang (*Rehmannia glutinosa* (Gaertn.) Libosch. ex DC.)	DNFB - induced AD in mice	Inhibited the activations of STAT3, MAPK and NF-κB signaling pathways and possessed a significant therapeutic effect on AD	Xiong et al. (2020)
Ta-Xi-San	Ku Shen (*Sophora flavescens* Aiton), Huang Bai (*Phellodendron amurense* Rupr.), Di Yu (*Sanguisorba officinalis* L.), Ma Chi Xian (*Portulaca oleracea* L.), Cang Shu (*Atractylodes lancea (Thunb.) DC.*), Bai Fan (*Alumen*)	DNCB - induced AD in mice	Via PI3K-Akt signaling pathway, MAPK signaling pathway, and TLR signaling pathway with the regulation of inflammatory response and transcription	Zhi et al. (2022)
BuShenYiQi Granule	Yin Yang Huo (*Epimedium brevicornum* Maxim), Huang Qi (*Astragalus mongholicus* Bunge), Di Huang (*Rehmannia glutinosa* (Gaertn.) Libosch. ex DC.)	OVA - induced AD in mice	Presented anti-inflam-matory and anti-allergic potential by systemically elevated levels of endogenous glucocorticoids and locally normalized function of skin HPA axis-like system	Kong et al. (2015)
Jiu-Wei-Yong-An Formula	Tu Fu Ling (*Smilax glabra* Roxb.), Di Huang (*Rehmannia glutinosa* (Gaertn.) Libosch. ex DC.), Che Qian Cao (*Plantago asiatica* L.), Ban Lan Gen (*Isatis tinctoria* L.), Lian Qiao (*Forsythia suspensa* (Thunb.) Vahl), Dang Gui (*Angelica sinensis* (Oliv.) Diels), Shan Yao (*Dioscorea oppositifolia* L.), Huang Shan Yao (*Dioscorea panthaica* Prain & Burkill), Ze Xie (*Alisma lanceolatum* With.)	DNCB - induced AD in mice	Attributed to blocking the JAK1/STAT3 and MAPK signaling pathways	Qinwufeng et al. (2022)
Pentaherbs formulaz	Jin Yin Hua (*Lonicera japonica* Thunb.), Bo He (*Mentha canadensis* L.), Huang Bai (*Phellodendron amurense* Rupr.), Mu Dan Pi (*Paeonia × suffruticosa* Andrews), Cang Shu (*Atractylodes lancea (Thunb.) DC.*)	OX - induced AD in mice	Suppress the releaseof pro-inflammatory cytokine IL-6 and chemokine CCL7 and CXCL8	Chu et al. (2020)

AD, atopic dermatitis; DNCB, 1-Chloro-2,4-dinitrobenzene; DNFB, 2,4-dinitrofluorobenzene; OVA, ovalbumin; OX, oxazolone; IL, interleukin.

#### 3.3.2 Single TCM botanical drug

**FIGURE 5:**
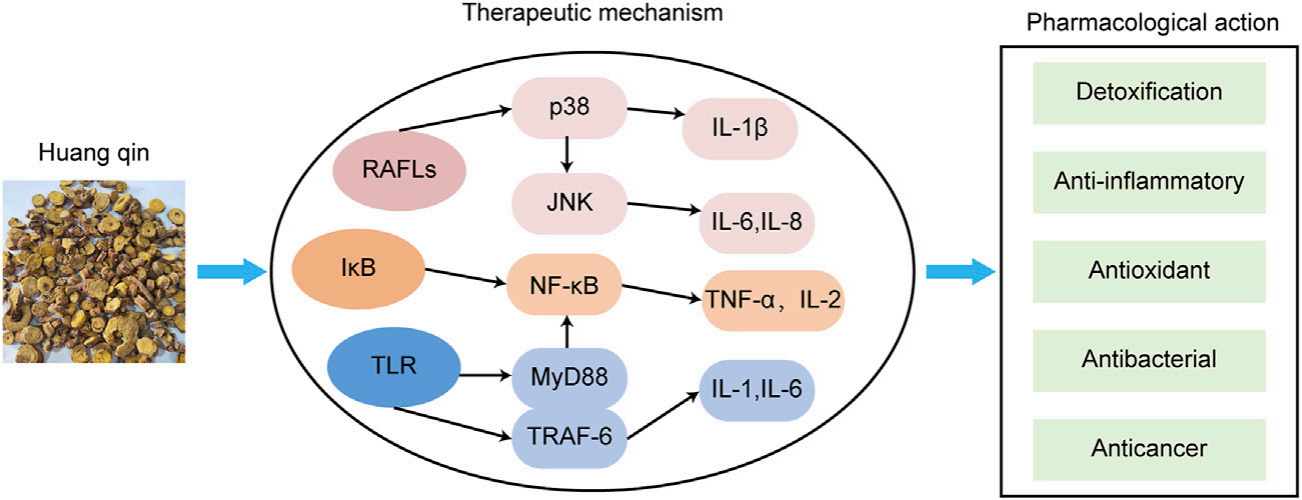
The main mechanisms of Huangqin against AD.

In addition to the above preparations, various individual botanical drug can also treat AD (Table 4). Lycopus lucidus Turcz has been shown to alleviate DNCB-induced AD in BALB/c mice. Scutellaria baicalensis Georgi (Huangqin) is commonly used to treat AD and has broad anti-inflammatory, immunosuppressive, and antibacterial functions (Wang L. et al., 2022; Lee and Im, 2023) (Figure 5).

**TABLE 4:** The effects of single TCM botanical drugs on AD.

Single botanical drugs	Evaluation model	The effect mechanism	The literature
He Zi (*Terminalia chebula* Retz.)	Dfe - induced AD in mice	Regulating anti-inflammatory factors *in vivo* and suppressing STAT1/3 and NF-κB signaling *in vitro*	Kim et al. (2022)
Bai Shao (*Paeonia lactiflora* Pall.)	DNCB - induced AD in mice	Increased the diversity of the gut microbiota and changed the microbial composition suppressing inflammatory cytokine production, inducing Foxp3 expression	Lee et al. (2022b)
Bai Xian (*Dictamnus dasycarpus* Turcz.)	DNFB - induced AD in mice	Inhibited AD-induced chronic itch, inflammation symptoms, epidermal thickening, in-flammatory cell infiltration, and downregulated the expression of MrgprA3 and TRPA1	Yang et al. (2022)
Bai zhi (*Angelica dahurica* (Hoffm.) Benth. & Hook.f. ex Franch. & Sav.)	MC903 - induced AD in mice	Decreased TRPV1 activity and protein expression in mice with inflammatory diseases	Zhu et al. (2022)
Zi Ding Xiang (*Syringa oblata* Lindl.)	DNCB - induced AD in mice	Down-regulatedthe expression of iNOS and COX-2, inhibiting the T cell-mediated allergic immune response	Fan et al. (2021)
Ji Xue Teng (*Spatholobus suberectus* Dunn.)	Dfe - induced AD in mice	Inhibits IFN-γ/TNF-α–Induced MAPK/STAT1/NF-κB	Song et al. (2022a)
She Chuang Zi (*Cnidium monnieri (*L.) Cusson)	OX - induced AD in mice	Inhibition of upregulation of p-Akt protein expression and downregulation of ZO-3 protein expression in the skin of AD model mice	Hu (2020)
Lu Hui (*Aloe vera* (L.) Burm.f.)	DNFB- induced AD in mice	Suppresses Th17 cell immune response, reduces IgE levels, and inhibits NF- κB signaling pathway	Chen et al. (2019)
Ku Shen (*Sophora flavescens* Aiton)	OVA- induced AD in mice	Regulate the expression of PAR-2 and downstream receptor protein TrK-A in skin lesions, and reduce the content of PGE2, LTB4, SP, and CGRP in serum	Song et al. (2022b)
Yi Yi Ren (*Coix lacryma-jobi var. ma-yuen* (Rom.Caill.) Stapf)	DNCB - induced AD in mice	Reduce the levels of IgE and IL-4 in serum, and increase IFN- γ Level, regulating the expression of AQP3, TLR2, and TLR4 in the skin	Wang (2018)
Hai Tong Pi (*Erythrina variegata* L.)	DNCB - induced AD in mice	Regulate p38/NF- κ B signaling pathways	Chen et al. (2023)
Zi Cao (*Lithospermum erythrorhizon* Siebold & Zucc.)	OX - induced AD in mice	Reduce the level of inhibitor protein IκBα, thereby inhibiting the expression of COX-2 and iNOS	Kim et al. (2008)

Dfe, Dermatophagoides farinae extract; AD, atopic dermatitis; DNCB, 1-Chloro-2,4-dinitrobenzene; DNFB, 2,4-dinitrofluorobenzene; OVA, ovalbumin; OX, oxazolone; MC903, Calcipotriol; IL, interleukin.

Research has shown that in addition to the aforementioned preparations, various individual TCM extracts also treat AD.

*Terminalia chebula* Retz. [Combretaceae] (HeZi, TC) is a botanical drug that belongs to a tree of the genus Elephantia in the family Meneraceae, and it is a dried and mature fruit. TC is commonly used as a TCM. (Li H. et al., 2022). The chemical metabolites isolated from TC are mainly divided into three types according to their structure: phenolic acids, tannins, and triterpenes. Most of the metabolites in TC have anti-inflammatory and antioxidant effects. As a result, TC single TCM may become a therapeutic drug for treating AD. Hye Jin Kim et al. used AD in induced by Dermatophagoides farinae extract (Dfe) in NC/Nga mice. The results showed that after treatment with TC reduced lesions caused by AD. TC treatment reduced the serum levels of histamine, IgE, and other chemokines, and can inhibit STAT1/3 and NF-κB signaling pathway by regulating inflammatory factors (Kim et al., 2022).

*Paeonia lactiflora* Pall. [Paeoniaceae] (BaiShao, PL) is a traditional botanical drug. Recently, several reports have shown that PL is effective for the treatment of various diseases (Ou et al., 2011; Zhang and Dai, 2012). Paeoniflorin and total paeoniflorin are the main metabolites of PL. Paeoniflorin has been shown to induce lymphocyte apoptosis (Tsuboi et al., 2004; Wu et al., 2019). The results of Lee et al. indicate that PL attenuates pathological changes in antibiotic-induced AD model mice (Lee S. Y. et al., 2022). Many studies have shown that white peony has anti-inflammatory and immunomodulatory activities (He and Dai, 2011). Shen et al. constructed 1-chloro-2,4-dinitrobenzene (DNCB) -induced mouse AD model and administered total peony glucosides (200 mg/kg) by gavage. Under the effect of total peony glucosides, the thickness of the ear, the score of the skin lesion degree, and the number of scratches were significantly reduced, and the damage to the skin structure compared with the model group, there was a significant decrease. Moreover, the infiltration of inflammatory cells and mast cells was reduced, and reduced the levels of IgE and IL-6, indicating that total peony glucosides can reduce the inflammatory reaction in AD model mice and improve the pathological symptoms of AD (Shen et al., 2021). Liu et al. treated AD model mice established with DNCB with total peony glycosides and found that total peony glycosides had an inhibitory effect on the symptoms of AD model mice. PL may be could inducing the expression of Foxp3, increasing the integrity of the intestinal barrier, and altering the composition of intestinal microbiota. TGP can significantly inhibit the activation of NLRP3, increase Th1-secreted IFN-γ levels, and inhibit Th2-secreted IL-4, thereby inhibiting the synthesis and expression of IgE and regulating the balance of Thl/Th2, producing a certain therapeutic effect on AD (Liu et al., 2017).

*Dictamnus dasycarpus* Turcz. [Rutaceae] (Bai Xian) also known as White Fresh Skin, Eight Stranded Cow, and Mountain Peony, is a perennial botanical drug in the Rutaceae family, belonging to the genus Lysimachia. The stem of Bai Xian is rigid and woody and grows to a height of up to 100 cm. In TCM theory, its effects are defined as reducing fever, detoxification, dispelling wind, and relieving itching. It can also treat skin itching, eczema, jaundice, rheumatism, and other diseases (Qin et al., 2021). It also has antitumour (Park et al., 2015), antioxidation (Lin et al., 2021), anti-inflammatory (Yang N. et al., 2023), antibacterial, and antiallergic effects (Qin et al., 2021). It can be taken orally in the form of a decocted liquid or used externally by grinding it into a powder. It can not only treat various diseases but also be used as a pollution-free pesticide (Du et al., 2023). Yang et al. used DNCB-induced AD mouse model to observe scratching behaviour and inflammatory behaviour and to evaluate the expression of MrgprA3 and TRPA1 in the skin and DRG. The data showed that dichloraz effectively inhibited AD-induced chronic itching, inflammatory symptoms, epidermal thickening, and inflammatory cell infiltration and downregulated the expression of MrgprA3 and TRPA1. Molecular docking also shows that fresh amine has a better binding affinity with MrgprA3. Dichloraz may inhibit chronic itching caused by AD through the histamine-independent itching pathway mediated by MrgprA3-TRPA1, indicating that dichloraz may have potential efficacy in the treatment of AD (Yang et al., 2022).

*Angelica dahurica* (Hoffm.) Benth. & Hook.f. ex Franch. & Sav. [Apiaceae](Bai Zhi) belongs to the Umbrellaceae genus and is a famous Chinese botanical drug. The roots of Angelica dahurica are often used to treat headaches, toothache, abscesses, boils and acne. They have functions such as alleviating disease symptoms, removing dampness, expelling pus, generating muscle, promoting blood circulation and relieving pain. Chen et al. used a gel with xanthopterin, an extract of Angelica dahurica, to treat mice with MC-903-induced AD-like skin. The experimental results showed that xanthopterin gel significantly improved the symptoms of AD in mice and significantly reduced the thickness of the mouse epidermal scales and serum IgE levels (Chen et al., 2022). The results of Zhu et al. showed that Bai Zhi also reduced activity and protein expression of TRPV1 (Zhu et al., 2022). Wang et al. used Angelica dahurica aloe gel to treat 16 patients with radiation dermatitis for 4 weeks. The final results showed that the clinical effect of Angelica dahurica aloe gel was equivalent to that of steroid drugs, which could help patients effectively improve skin pain and itching, and no adverse reactions occurred (Wang and Lei, 2018).

*Cnidium monnieri* (L.) Cusson [Apiaceae] (She Chuang Zi) is the mature and dried fruit of the Apiaceae plant Cnidii. Osthol (OS) in Cnidii is a natural coumarin with a long medicinal history and can be used for a variety of diseases, both internally and externally, with excellent external treatment and multiple effects, such as anti-inflammatory, antiviral and antiallergic effects. Kordulewska et al. used osthol to treat 3D skin models of AD stimulated by LPS and histamine *in vitro*, and the results showed that osthol significantly improved the integrity of the 3D skin models and reduced the secretion of pro-inflammatory cytokines, chemokines, and proteins (Kordulewska et al., 2021). Chen et al. intraperitoneally injected mice with osthol, and the skin lesions of the mice were significantly improved. The expression of connexin mRNA was abnormally downregulated, while the expression of p-Akt was downregulated in mice after treatment with osthol. The expression of connexin mRNA was abnormally downregulated, with ZO-3 being the most significantly downregulated. Moreover, the use of osthol downregulated the expression of p-Akt, indicating that osthol can regulate the expression of tight junction proteins in the skin of AD model mice through the PI3K/Akt pathway, which can improve skin barrier damage (Chen J. et al., 2021). Du et al. pointed out that osthol has strong active oxygen scavenging and anti-inflammatory effects, and its anti-inflammatory activity is mediated through a variety of mechanisms, including inhibition of various transcription factors, and downregulation of a variety of pro-inflammatory factors and chemokines (Du et al., 2020).

#### 3.3.3 TCM metabolites

In recent years, with the advancement of science, many researchers have conducted detailed research on the active metabolites of TCM and identified many plant metabolites.

Piceatannol is a natural polyphenol metabolite found in a variety of edible plants (blueberries, grapes, passionfruit, etc.). It has a variety of health promotive functions, such as anti-diabetes, neuroprotection, anti-allergy, and anti-ageing activities (Yang W. et al., 2023). Lee et al. successfully reduced the symptoms of AD, by using a treatment drug supplemented with piceatannol in an AD mouse model induced by dust mite extract (Lee C. H. et al., 2022). Yang used network pharmacology and cytology experiments to demonstrate that piceatannol has an inhibitory effect on the expression of NF-κB and that NF-κB is an effective target for the treatment of AD with piceatannol, providing a new possibility for the treatment of AD. Piceatannol inhibited the infiltration of immune cells into the skin of DFE-induced AD model mice, reducing TNF α/IFN-γ levels, and induced phosphorylation of JAK STAT protein in HaCaT cell lines. The results of Macromolecular docking study showed that there was a strong interaction between piceatannol and JAK1 (Yang, 2022).

Vasicine is a pyrrolo [2,1-b] quinazoline alkaloid isolated from the flower of duckbill. Duckbill alkaloids have many active sites and exhibit a wide range of biological activities after binding to receptors, and have anti-inflammatory, antiasthmatic, antioxidant, antitussive and low toxicity characteristics (Kong et al., 2022). Zhang et al. evaluated the anti-AD effect of duckbill alkaloids induced by DNCB in mice. The potential anti-allergic effect of duckbill alkaloids was evaluated using a passive skin anaphylaxis (PCA) test. They found that oral administration of duckbill alkaloids could reduce histopathological changes and restore epidermal thickness, improving the severity of AD-like lesions in the skin. Duckbill alkaloids can significantly inhibit mast cell infiltration and effectively reduce serum IgE and Th2 cytokine levels, demonstrating their therapeutic potential for AD. In a PCA mouse model, duckbill alkaloids reduced serum IgE levels (Zhang et al., 2022a). This result suggests duckbill alkaloids may benefit allergic or atopic diseases associated with elevated IgE levels (Table 5).

**TABLE 5:** The effect of TCM metabolites on AD.

Name	Botanical drug	Evaluationmodel	Mechanism of action	The literature
Piceatannol	*Reynoutria japonica* Houtt.	Dfe - induced AD in mice	Downregulation of inflammatory markers, including serum and skin TARC and MDC. Piceatannol decreased phosphorylation of JAK-STAT protein	Lee et al. (2022a)
Vasicine	*Justicia adhatoda* L.	DNCB - induced AD in mice	Inhibited the infiltration of mast cells in the skin and reduced the levels of pro-Th2 and Th2 cytokines as well as immunoglobulin E in the serum. Finally, vasicine inhibited the expression of pro-Th2 and Th2 cytokines in skin tissues	Zhang et al. (2022a)
Myricetin	NA	MC903 - induced AD in mice	Blocking the NF-κB and STAT1 signal pathway	Hou et al. (2022a)
Quercetin	NA	MC903 - induced AD in mice	Reduce the expression of CCL17, CCL22, IL-4, IL-6, IFN-γ and TNF-α	Hou et al. (2019)
Scutellarein	*Erigeron breviscapus* (Vaniot) Hand.-Mazz.	DNFB - induced AD in mice	Inhibiting TRPV3	Wang et al. (2022b)
(-) -α-Bisabolol	*Matricaria chamomilla* L.	DNCB - induced AD in mice	Inhibiting MAPK and NF-κB Signaling in MC	Li et al. (2022a)
Phellopterin	*Glehnia littoralis* (J.G.Cooper) F.Schmidt ex Miq.	MC903 - induced AD in mice	Phellopterin suppressed phosphorylation of signal transducer and STAT3 at Tyr705, and the expression of TSLP and IL-33 in epidermal keratinocytes of AD-like lesions	Chen et al. (2022)
Notoginsenoside R1	*Panax notoginseng* (Burkill) F.H.Chen	LPS - establish the *in vitro* cell	Inhibiting inflammation through suppressing the NF-κB signaling pathway and NLRP3 inflammasome activation	Wang and Ma (2019)
7-Methoxyisoflavone	NA	OX - induced AD in mice	Regulating Th1/Th2 balance	Dong et al. (2022)
Pseudoephedrine	*Ephedra sinica* Stapf	DNCB - induced AD in mice	Suppressed the activation of MAPKs and NF-κB signaling pathways *in vivo* and *in vitro*	Chen et al. (2020)
Daphnetin	*Daphne kamtschatica var. kamtschatica*	DNCB - induced AD in mice	Decreased the expression levels of histamine, IL-4, IL-6, IL-13, MIP-1α and TNF-α, and reduced the protein expression levels of phosphorylated MAPKs, P-Lyn and P-syk in the RBL-2H3 cells	Zhang et al. (2022b)
*Lonicera japonica* polysaccharide	*Lonicera japonica* Thunb.	DNCB - induced AD in mice	By promoting Nrf2 activation and NLRP3 inflammasome degradation via p62	Bai et al. (2023)
2,4-dimethoxy-6-methylbenzene-1,3-diol	*Antrodia camphorata*	OVA - induced AD in mice	Stopped the upregulation of chemokines (CCL5 and CCL17) and increased the expression of differentiation proteins (filaggrin, involucrin, and integrin β-1)	Yang et al. (2018)
Paeonol	*Paeonia × suffruticosa* Andrews	DNCB - induced AD in mice	Reduced the protein expression levels of p-p38 and p-ERK	Meng et al. (2019)
Bisdemethoxycurcumin	*Zingiber officinale* Roscoe	DNCB - induced AD in mice	Inhibit the mRNA expression levels of chemokines and inflammatory cytokines and the activation of the MAPK and NF-κB signaling pathways	Wang et al. (2022c)
Crude polysaccharide	*White wax scale*	DNCB - induced AD in mice	Through modulating T cell-elicited immune responses and CD4^+^T cell polarization	Lin et al. (2017)
Glycyrrhizic acid	Glycyrrhiza glabra L.	MC903 - induced AD in mice	Suppressed the Th1/Th2/Th17-immune responses in the dLNs, inhibited the migration of LCs in dLNs	Hou et al. (2022b)
Rutaecarpine	*Tetradium ruticarpum* (A.Juss.) T.G.Hartley	DNFB- induced AD in mice	Regulation of IL-4/STAT6 signaling pathway	Dun et al. (2022)
Isoglycyrrhizin	*Glycyrrhiza glabra* L.	DNCB- induced AD in mice	Reduce serum IL-4 and TNF- α And IgE levels, inhibiting the JAK-STAT pathway to mediate	Li et al. (2021b)
Berberine tannate	*Coptis chinensis* Franch.	DNCB- induced AD in mice	Suppress participation in PPAR-γ Upregulated HMGB1, RAGE, NF-κB, COX-2 signaling pathway and inflammatory cytokines such as IL-1β, TNF-α, IL-4 and IFN-γ	Ma et al. (2023)

NA, not applicable; Dfe, Dermatophagoides farinae extract; AD, atopic dermatitis; DNCB, 1-Chloro-2,4-dinitrobenzene; DNFB, 2,4-dinitrofluorobenzene; OVA, ovalbumin; OX, oxazolone; MC903, Calcipotriol; LPS, lipopolysaccharide; IL, interleukin.

### 3.4 Clinical evidence for the use of TCM in AD

Currently, many TCM treatment methods have been used for the clinical treatment of AD, and many TCMs for AD treatment have entered clinical research (reference website:https://www.yaozh.com, Table 6). Controlled studies of AD are often compared to glucocorticoids, calcineurin inhibitors, antibiotics and antihistamines.

**TABLE 6:** Clinical trials of TCM on AD.

Stage	Drug name	Metabolites	Method	Intervention measures		Indication
				Experimental group	Control group	
Phase II	Peitu Qingxin Granules	*Atractylodes macrocephala* Koidz., *Forsythia suspensa* (Thunb.) Vahl, *Hydrastis canadensis* L., *Pseudostellaria heterophylla* (Miq.) Pax, *Coix lacryma-jobi* L., *Dioscorea oppositifolia* L., *Concha Margaritifera*, *Dictamnus dasycarpus* Turcz., *Glycyrrhiza glabra* L.	Random double-blind	PeiTu QingXin Granules	PeiTu QingXin Granules	AD
Exploratory study/pre- test	Zicao Ointment	*Arnebia euchroma* (Royle ex Benth.) I.M.Johnst., *Angelica dahurica* (Hoffm.) Benth. & Hook.f. ex Franch. & Sav., *Rehmannia glutinosa* (Gaertn.) Libosch. ex DC., *Saposhnikovia divaricata* (Turcz. ex Ledeb.) Schischk., *Angelica sinensis* (Oliv.) Diels	Randomized parallel control	Zicao Ointment	Mometasone furoate	AD
Exploratory study/pre- test	Taxisan	*Rhus chinensis* Mill., *Zanthoxylum armatum* DC., *Cnidium monnieri* (L.) Cusson, *Sophora flavescens* Aiton, *Alumen*, *Allium cepa* L.	Randomized parallel control	Taxisan	Moisturizer (Vaseline, glycerin and paraffin oil)	AD
NA	Anshenjianpizhiyang granule	NA	Randomized parallel	Anshenjianpizhiyang granule	placebo	Mild to moderate AD
Exploratory study/pre- test	Jiuwei Yongan Tang Granules	*Alumen*, *Dioscorea oppositifolia* L., *Forsythia suspensa* (Thunb.) Vahl, *Isatis tinctoria subsp. tinctoria*, *Dioscorea collettii var. hypoglauca* (Palib.) S.J.Pei & C.T.Ting, *Rehmannia glutinosa* (Gaertn.) Libosch. ex DC., *Angelica sinensis* (Oliv.) Diels, *Alisma plantago-aquatica subsp. orientale* (Sam.) Sam., P*lantago ovata* Forssk.	Randomized parallel	Jiuwei Yongan Tang Granules	Xianteming	AD
Exploratory study/pre- test	Qinzhu Liangxue granule	*Scutellaria baicalensis* Georgi, *Margaritifera Concha*, *Rehmannia glutinosa* (Gaertn.) Libosch. ex DC., *Scrophularia ningpoensis* Hemsl., *Saposhnikovia divaricata* (Turcz. ex Ledeb.) Schischk., *Lithospermum erythrorhizon* Siebold & Zucc., *Glycyrrhiza glabra* L., *Vincetoxicum mukdenense* Kitag., *Paeonia lactiflora* Pall., *Paeonia × suffruticosa* Andrews, *Lonicera japonica* Thunb.	Randomized parallel	Qinzhu Liangxue granule	Mizolastine Sustained Release Tablets	AD
NA	Chushi Zhiyang Mixture	*Lonicera japonica* Thunb, *Scutellaria baicalensis* Georgi, *Rehmannia glutinosa* (Gaertn.) Libosch. ex DC., *Paeonia lactiflora* Pall., *Bassia scoparia* (L.) Beck, *Poria Cocos* (Schw.) Wolf.	Randomized parallel	Chushi Zhiyang Mixture + Hydrocortisone butyrate cream	Cetirizine + Hydrocortisone butyrate cream	Mild to moderate AD
Phase IV	Bu Shen Yi Qi Fang	*Astragalus mongholicus* Bunge, *Rehmannia glutinosa* (Gaertn.) Libosch. ex DC., *Epimedium brevicornum* Maxim, etc	Randomized parallel	Bu Shen Yi Qi Fang + Desloratadine tablet	Imitation of Bu Shen Yi Qi Fang + Desloratadine tablet	Severe AD (Patients with syndrome of kidney deficiency and qi deficiency)
Exploratory study/pre- test	Chuankezhi	*Epimedium sagittatum* (Siebold & Zucc.) Maxim., *Gynochthodes officinalis* (F.C.How) Razafim. & B.Bremer	Randomized parallel	Basic treatment + Chuankezhi	Basic treatment	AD (Yin deficiency syndrome)

NA, not applicable; AD, atopic dermatitis.

At the same time, the clinical efficacy of some TCM was also noted (Table 7). For example, Geng and Zhang (2023) conducted a clinical study on the efficacy of orally administered Danggui Zhiyang Formula (prescription composition: Angelica sinensis, Ligusticum chuanxiong, Radix Rehmanniae, Stir-fried white peony, *S. divaricata* (Turcz. ex Ledeb.) Schischk., Stir-fried tribulus, bergamot, Zhetong bark, astragalus, raw liquorice) on AD. The effective rate was 91.89%, which was significantly larger than the control group (61.76%).

**TABLE 7:** Clinical study of TCM on AD in literature.

Drug name	Control group	Group prescription	Number ofcases	Effective rate	Control group	Reference
Experience group				Experience group		
Maidong Dendrobium lotion + Desloratadine tablets	Desloratadine tablets	*Dendrobium officinale* Kimura & Migo, *Ophiopogon intermedius* D.Don, *Bletilla striata (*Thunb.) Rchb.f., *Portulaca oleracea* L., *Phellodendron amurense* Rupr., *Phaseolus vulgaris* L., *Angelica sinensis* (Oliv.) Diels, *Rehmannia glutinosa* (Gaertn.) Libosch. ex DC., *Glycyrrhiza glabra* L., *Linum usitatissimum* L., *Prunus persica* (L.) Batsch, *Carthamus tinctorius* L.	40	79.95%	45.74%	Liu and Sun (2023)
Danggui Zhiyang Formula	Runzao Zhiyang Capsule	*Angelica sinensis* (Oliv.) Diels, *Conioselinum anthriscoides* ‘Chuanxiong’, *Rehmannia glutinosa* (Gaertn.) Libosch. ex DC., *Plumbago zeylanica* L., *Saposhnikovia divaricata* (Turcz. ex Ledeb.) Schischk., *Tribulus terrestris* L., *Citrus × limon* (L.) Osbeck, *Euphorbia pekinensis* Rupr., *Astragalus mongholicus* Bunge, *Glycyrrhiza glabra* L.	70	91.89%	61.76%	Geng and Zhang (2023)
Addition and subtraction of Sijunzi Decoction and Daochi Powder	Loratadine Syrup + Hydrocortisone Butyrate Ointment	*Pseudostellaria heterophylla* (Miq.) Pax, *Atractylodes macrocephala* Koidz., *Poria Cocos* (Schw.) Wolf., *ehmannia glutinosa* (Gaertn.) Libosch. ex DC., *Lophatherum gracile* Brongn., *Portulaca oleracea* L., *Scutellaria baicalensis* Georgi, *Bauhinia × blakeana* Dunn, *Bassia scoparia* (L.) Beck, *Glycyrrhiza glabra* L.	80	95.24%	88.10%	Ceng et al. (2022)
Lingshu Qushi Decoction Combined with Dry Branch Ear Position + Tacrolimus ointment	Loratadine Tablets + Tacrolimus ointment	*Poria Cocos* (Schw.) Wolf., *Atractylodes macrocephala* Koidz., *Codonopsis pilosula* (Franch.) Nannf., *Lophatherum gracile* Brongn., *Platycodon grandiflorus* (Jacq.) A.DC., *Phragmites australis* (Cav.) Trin. ex Steud., *Citrus reticulata* Blanco, *Magnolia officinalis* Rehder & E.H.Wilson, *Wurfbainia villosa* (Lour.) Škorničk. & A.D.Poulsen, *Bassia scoparia* (L.) Beck, *Coix lacryma-jobi* L.,*Atractylodes macrocephala* Koidz., *Glycyrrhiza glabra* L.	56	92.31%	69.23%	Gao (2022)
Fu Ling Tai Bai Zhi Yang Decoction + Mucopolysaccharide Polysulfate Cream	Mucopolysaccharide Polysulfate Cream	*Atractylodes macrocephala* Koidz., *Poria Cocos* (Schw.) Wolf., *Pseudostellaria heterophylla* (Miq.) Pax, *Dioscorea oppositifolia* L., *Bassia scoparia* (L.) Beck, *Perilla frutescens* (L.) Britton, *Alisma plantago-aquatica subsp. orientale* (Sam.) Sam., *Angelica sinensis* (Oliv.) Diels, *Platycodon grandiflorus* (Jacq.) A.DC., *Glycyrrhiza uralensis* Fisch. ex DC.	70	80%	34.3%	Shen (2022)
Huaiqi Yellow Granules + Fluticasone propionate cream,+desloratadine tablets	Fluticasone propionate cream + desloratadine tablets	*Vicia faba* L., *Lycium barbarum* L., *Polygonatum odoratum* (Mill.) Druce	60	96.67%	73.3%	Liu (2022)
Long Mu Jia Wei Decoction	Loratadine Tablets	*Os Draconis*, *Concha Ostreae Calcinationis*, *Forsythia suspensa* (Thunb.) Vahl, *Pastinaca sativa* L., *Ephedra sinica* Stapf, *Poria Cocos* (Schw.) Wolf.	60	90.3%	76.67%	Chi et al. (2021)
Yangxue Qufeng Decoction	Bastine tablets	*Paeonia lactiflora* Pall., *Reynoutria japonica* Houtt., *Sesamum indicum* L., *Tribulus terrestris* L., *Angelica sinensis* (Oliv.) Diels, *Saposhnikovia divaricata* (Turcz. ex Ledeb.) Schischk. ,*Bombyx mori Linnaeus*, *Conioselinum anthriscoides* ‘Chuanxiong’, *Cicadae Periostracum*	95	95.83%	80.85%	Xiao and Zhao (2021)
Yupingfeng Granules + Baiji Polysaccharide + Fu’an Xiaojiao	Yupingfeng Granules + Baiji Polysaccharide + Zinc Oxide	Fu’an Xiaojiao Lotion: Tarpaulin, *Chrysanthemum indicum* L., *Poria Cocos* (Schw.) Wolf., *Alumen*, and *Cnidium monnieri* (L.) Cusson. Yupingfeng Granules: *Astragalus mongholicus* Bunge, *Atractylodes macrocephala* Koidz., *Saposhnikovia divaricata* (Turcz. ex Ledeb.) Schischk.	156	100%	84.6%	Chen et al. (2021a)
Qiwei Baizhu Powder	Cetirizine hydrochloride drops	*Pseudostellaria heterophylla* (Miq.) Pax, *Dolomiaea costus* (Falc.) Kasana & A.K.Pandey, *Poria Cocos* (Schw.) Wolf., *Atractylodes macrocephala* Koidz., *Agastache rugosa* (Fisch. & C.A.Mey.) Kuntze, Pueraria lobata, *Glycyrrhiza glabra* L.	80	86.84%	67.57%	Hong (2021)
Shenling Baizhu Powder Modified Granules	Mometasone Furoate Cream	*Codonopsis pilosula* (Franch.) Nannf., *Poria Cocos* (Schw.) Wolf., *Atractylodes macrocephala* Koidz.,*Vicia lens* (L.) Coss. & Germ., *Citrus reticulata* Blanco, *Dioscorea oppositifolia* L., *Glycyrrhiza glabra* L., *Nelumbo nucifera* Gaertn., *Coix lacryma-jobi* L., *Platycodon grandiflorus* (Jacq.) A.DC., *Ziziphus jujuba* Mill.	60	76.7%	46.7%	Liu (2021)

### 3.5 Advantages and disadvantages of TCM in the treatment of AD

AD, a multifactorial inflammatory skin disorder, necessitates therapeutic strategies that address both symptomatic relief and long-term disease modulation. While modern medical treatments, such as topical corticosteroids and biologics, prioritize rapid suppression of inflammation, TCM emphasizes holistic regulation of immune dysregulation, skin barrier repair, and systemic balance. To elucidate their distinct profiles, a comparative evaluation of TCM and modern medicine is essential, focusing on efficacy, safety, mechanisms, and practicality. The Table 8 summarizes their respective advantages and limitations in AD management.

**TABLE 8:** Comparison between TCM and modern medicine.

Aspect	TCM	Modern medical
Speed of efficacy	Gradual (weeks to months)	Rapid (days to weeks)
Mechanistic focus	Multi-target (anti-inflammatory, barrier repair, immune modulation)	Targeted (IL-4/IL-13 inhibition, JAK-STAT blockade)
Anti-inflammatory action	Broad, multi-target (cytokines, barrier repair)	Targeted (IL-4/IL-13 inhibitors)
Side effects	Generally mild; risk of botanical drug interactions or contaminants	Common (skin atrophy, immunosuppression, rebound flares)
Prevention of Relapse	Emphasizes systemic balance (Root Treatment)	Limited to symptom control; recurrence common post-discontinuation
Personalization	Tailored to individual constitution and syndrome differentiation	Standardized protocols with limited customization
Cost-effectiveness	Lower long-term costs (botanical drug formulations)	High
Evidence base	Growing preclinical data; limited large-scale RCTs	Robust RCTs and regulatory validation

TCM, traditional Chinese medicine; RCT, randomized controlled trial.

## 4 Conclusion and prospects

TCM has shown promising potential in the treatment of AD. This article confirms the important therapeutic effect of TCM on AD symptoms from both research and clinical studies.

The pathogenesis of AD involves the complex interaction of multiple cell types and genetic and environmental factors and is not fully understood. Although topical glucocorticoid therapy has a certain clinical effect on AD, it has a high recurrence rate, adverse reactions are difficult to avoid, and the long-term treatment effect is not ideal. In recent years, as research on TCM and its clinical applications has progressed, it has been found that TCM plays an increasingly important role in the treatment of AD, with significant curative effects, few side effects and a low recurrence rate. TCM and certain preparations can play a therapeutic role through corresponding mechanisms regardless of dosage and route of administration. The treatment of atopic dermatitis using TCM is characterized by targeting multiple pathways and multiple targets, and it demonstrates significant therapeutic effects.

Although there are still some issues in existing research, the clinical efficacy of TCM in treating AD is evident. To enhance the effectiveness of TCM in treating AD, we should delve deeper into the correlation between various mechanisms and indicators. The mechanism of action of a drug refers to the reasons why the tested drug exerts relevant effects on the body. This index represents the response of the subject after administration. By observing this index, we analyzed the internal causes that led to changes in this index, determined the internal relationships between these indicators, and explored their mechanisms of action. Integrating the advantages of TCM and Western medicine, along with the principles of TCM syndrome differentiation, may enhance efficacy and reduce adverse reactions. This integrated approach leverages the strengths of both medical traditions, potentially leading to more effective and safer treatment options for AD patients.
